# Renin–angiotensin system inhibitors positively impact on multiple aging regulatory pathways: Could they be used to protect against human aging?

**DOI:** 10.14814/phy2.16094

**Published:** 2024-06-26

**Authors:** Elena M. V. de Cavanagh, Felipe Inserra, León Ferder

**Affiliations:** ^1^ Department of Research Instituto Massone SA Buenos Aires Argentina; ^2^ Department of Medicine Maimonides University Buenos Aires Argentina; ^3^ Master of Vascular Mechanics and Arterial Hypertension, Postgraduate Department Austral University Pilar Argentina

**Keywords:** antiaging, mitochondria, RAS‐blockers

## Abstract

The renin–angiotensin system (RAS)—a classical blood pressure regulator—largely contributes to healthy organ development and function. Besides, RAS activation promotes age‐related changes and age‐associated diseases, which are attenuated/abolished by RAS‐blockade in several mammalian species. RAS‐blockers also increase rodent lifespan. In previous work, we discussed how RAS‐blockade downregulates mTOR and growth hormone/IGF‐1 signaling, and stimulates AMPK activity (together with klotho, sirtuin, and vitamin D‐receptor upregulation), and proposed that at least some of RAS‐blockade's aging benefits are mediated through regulation of these intermediaries and their signaling to mitochondria. Here, we included RAS‐blockade's impact on other aging regulatory pathways, that is, TGF‐ß, NF‐kB, PI3K, MAPK, PKC, Notch, and Wnt, all of which affect mitochondria. No direct evidence is available on RAS/RAS‐blockade‐aging regulatory pathway–mitochondria interactions. However, existing results allow to conjecture that RAS‐blockers neutralize mitochondrial dysfunction by acting on the discussed pathways. The reviewed evidence led us to propose that the foundation is laid for conducting clinical trials aimed at testing whether angiotensin‐converting enzyme inhibitors (ACEi) or angiotensin receptor blockers (ARB)—even at subclinical doses—offer the possibility to live longer and in better health. As ACEi and ARB are low cost and well‐tolerated anti‐hypertension therapies in use for over 35 years, investigating their administration to attenuate/prevent aging effects seems simple to implement.

## Graphical abstract

Aging is accompanied with changes in (i) signaling pathways that regulate the aging process, that is, TOR, AMPK, insulin/IGF‐1, TGF‐beta, NF‐kB, PI3K, MAPK, PKC, Notch and Wnt; (ii) activity and responsiveness of the renin–angiotensin system (RAS); (iii) epigenetic modulation of gene expression. These changes negatively impact on mitochondrial function, resulting in tissue damage and loss of function. RAS contributes to aging by promoting (i–iii). ACEi/ARB (RAS‐blockers)—even at subclinical levels—can block/attenuate (i–iii) thereby reducing age‐related decline. Well‐designed clinical trials would clarify whether long‐term low‐level RAS‐blockade can prolong human life and reduce age‐associated disease. ROS, reactive oxygen species; MDP, mitochondria‐derived peptides.

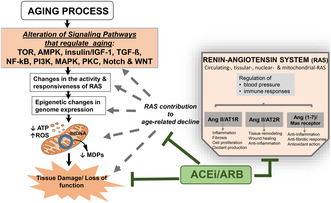

## 1 AGING AND MITOCHONDRIA

Aging is characterized by a broad deterioration of cellular function owing to the accumulation of damaged biomolecules and organelles. Mitochondria were once viewed as essential organelles committed exclusively to cellular energy production; nonetheless, nowadays they are also known to strongly contribute to the regulation of calcium homeostasis, tissue oxygen gradients, cell apoptosis, synthesis of metabolic precursors, and intracellular signaling. However, as a result of partial oxygen reduction by the mitochondrial respiratory chain, these organelles are crucial cellular reactive oxygen species (ROS) sources and are themselves targets of ROS‐induced injury. In this scenario, a plausible consequence of mitochondrial function derangement induced by harmful stimuli is the progression toward sickness and aging [for a recent thorough review please refer to Harrington et al. (2023)]. Thus, the mitochondrial free radical theory of aging (Harman, 1972; Miquel et al., 1980) proposes that mitochondrial ROS, generated in the course of normal metabolism, lead to the accumulation of oxidative damage which ultimately results in age‐associated changes.

It follows that interventions aimed at protecting from mitochondrial decay are expected to restrict the development of age‐related disease and general age‐associated deterioration.

## 2 THE RENIN–ANGIOTENSIN SYSTEM

### 2.1 The circulating‐, tissular‐, nuclear‐, and mitochondrial‐RAS

The classic understanding of the renin–angiotensin system (RAS) considers that its main function is the regulation of blood pressure, which is achieved by managing water and electrolyte balance, as well as by exerting vasomotor and trophic actions. However, this essential action has overshadowed other important effects of the RAS, including the promotion of oxidant production, inflammation, cell proliferation, and fibrosis (Forrester et al., 2018), and the regulation of immune responses (Hoch et al., 2009).

Briefly, the RAS is composed of the protein angiotensinogen, which after being released to the circulation by the liver is cleaved into angiotensin I (Ang I) by the kidney‐derived protease known as renin; this is followed by pulmonary angiotensin‐converting enzyme (ACE)‐mediated cleavage of Ang I to generate angiotensin II (Ang II), the main effector of the RAS. Ang II exerts its actions by binding to Ang II type 1 receptors (AT1R) or Ang II type 2 receptors (AT2R). Activation of AT1R by Ang II mainly promotes the regulation of blood pressure, whereas AT2R primarily participate in tissue remodeling and wound healing. Also, after binding to its AT1R, Ang II stimulates the generation of cytoplasmic and mitochondrial ROS, as well as activation of redox‐sensitive signaling cascades (Touyz, 2003).

The RAS includes a counter regulatory system (Paz Ocaranza et al., 2020) that opposes the above mentioned cardiovascular, pro‐inflammatory and pro‐fibrotic actions, and comprises angiotensin‐converting enzyme 2 (ACE2) and neprilysin (NEP) that can cleave Ang II and Ang I, respectively, into angiotensin 1–7 [Ang (1–7)], where ACE2 is potentially the preferred pathway for Ang (1–7) formation (Chappell, 2016); in addition, ACE2 cleaves Ang I into angiotensin 1–9 [Ang (1–9)] that mediates vasodilation and the reduction of blood pressure after activating AT2R, which stimulates nitric oxide (NO) generation and natriuresis (Ocaranza et al., 2014).

After binding to the Mas receptor, Ang (1–7) induces vasodilation, lowering of both blood pressure and fibrosis, among other effects (Passos‐Silva et al., 2013; Tallant et al., 2005). Ang (1–7)'s beneficial actions were found to be associated with the activation of phosphatases, such as Src homology 2‐containing protein tyrosine phosphatase [SHP]‐1 (Gava et al., 2009), the phosphoinositide phosphatase and tensin homolog (PTEN) (Modgil et al., 2012), and dual specificity protein phosphatase 1 (DUSP‐1) (Cook et al., 2010). As a whole, this evidence indicates that regulation of the activity of cell phosphatases is one of the key strategies by which Ang (1–7) contributes to an array of biological actions.

Interestingly, Ang (1–7) was also found to exert important antioxidant actions by variously decreasing NADPH oxidase (NOX) mRNA levels (Liu, Lv, et al., 2012), attenuating the expression of oxidative stress‐induced proteins (i.e. heme oxygenase‐1, NOX4, and NRF2) (Shi et al., 2015), and reducing ROS generation (Shi et al., 2015), among others. In cultured cardiomyocytes, the beneficial effects of Ang (1–7) against Ang II‐induced oxidant stress were shown to be mediated by increased mitochondrial MnSOD expression, which resulted from Sirt3‐signaling activation (Guo et al., 2017).

In this setting, it is important to note that the most frequently prescribed RAS‐blockers [angiotensin‐converting enzyme inhibitors (ACEi) and Ang II receptor blockers (ARB)] are aimed at inhibiting the canonical RAS pathway, consequently redirecting RAS peptides to the noncanonical pathway and supplying agonists for AT2R and Mas receptor activation (Re, 2004).

In addition to the circulating RAS, a variety of tissues display local RAS components that participate in the regulation of organ functions (Bader, 2010; Re, 2004). Of note, RAS‐blockers (ACEi and ARB) display diverse beneficial effects, which were convincingly shown to chiefly result from inhibition of tissue RAS (Saravi et al., 2021). Furthermore, both AT1R and AT2R were identified within mitochondria (Abadir et al., 2011), where AT2R expression prevails over that of AT1R, and mitochondrial AT2R activation stimulates NO production thereby inhibiting state 3 respiration, and pointing to a role for mitochondrial RAS in the modulation of mitochondrial energy production. Regarding the origin of mitochondrial NO, the study authors acknowledged that the existence of an inner membrane mitochondrial nitric oxide synthase (mtNOS) is under strong debate. However, as the observed increase of NO production in response to an AT2R agonist occurred in isolated mitochondria, and AT2R were detected in the inner mitochondrial membrane, the results may be interpreted to endorse the presence of mtNOS. In this scenario, abundant reports have identified NO within mitochondria. Several sources of intra‐mitochondrial NO have been proposed, including the controversial mtNOS, diffusion of extra‐mitochondrial NO into the organelle after being produced in the cytoplasm by other NOS isoforms (either neuronal NOS, inducible NOS, or endothelial NOS), and finally, NO produced inside mitochondria through enzymatic reactions that do not involve L‐arginine metabolism by NOS (Lacza et al., 2009). Importantly, Abadir et al (Abadir et al., 2011) found that in kidney tubular cells from aging mice the expression of mitochondrial AT2R was lower, and that of mitochondrial AT1R was higher, than those observed in young mice; in addition, long‐term ARB (losartan) treatment annulled mitochondrial AT2R age‐related changes and modestly abated mitochondrial AT1R expression. These observations harmonize with reports showing that ARB curtail age‐associated mitochondrial dysfunction (de Cavanagh et al., 2003). It has been proposed that mitochondrial Ang II receptors are activated by Ang II generated by cellular RAS within the cytoplasm; alternatively, another candidate to activate mitochondrial AT2R is des‐aspartyl1‐Ang II (Ang III)—an Ang II metabolite with higher lipophilicity than its parent compound (Abadir et al., 2011).

Adding an extra level of complexity to the RAS, AT1R, AT2R, and Mas receptors were identified in the nucleus of renal cells; also, nuclear angiotensin receptors were reported in hepatocytes, neurons, lymphocytes, and cardiac cells [reviewed in Gwathmey et al. (2012)].

Evidence obtained in sheep proximal tubules, indicates the presence of enzymatically active nuclear ACE, ACE2, and neprilysin, that were able to generate Ang II from Ang I, Ang (1–7) from Ang II, and Ang (1–7) from Ang I, respectively. Also, in isolated renal cortical nuclei, Ang II stimulated the generation of both ROS—known to be produced after AT1R activation—and NO after the activation of either AT2R or Mas receptor (Gwathmey et al., 2011). Therefore, as proposed by Gwathmey et al. (2012), the opposing intracellular AT1R and Ang (1–7)/Mas receptor pathways may play key roles inside cells by acting within nuclei or other intracellular compartments by way of regulating ROS, NO, and calcium levels that are known to affect gene expression. Interestingly, isolated sheep kidney nuclei were shown to express endothelial NO synthase (eNOS), in addition to soluble guanylate cyclase which is the main receptor for NO (Gwathmey et al., 2009).

Summarizing, the above information points to the concept that ACEi and ARB, by blocking the cell/tissue deteriorating actions of the ACE–Ang II–AT1R axis of the circulating‐, tissular‐, nuclear‐, and/or mitochondrial‐RAS—thus diverting peptides to the protective Ang II/Ang III‐AT2R axis and the Ang (1–7)/Mas receptor pathway—may protect against age‐related decay.

### 2.2 The RAS and regulation of immune responses

Apart from its key role in the regulation of blood pressure by the RAS, ACE is also involved in both innate and adaptive immune responses through the modulation of macrophage and neutrophil function. ACE overexpression in several immune cell types leads to improved bactericidal and antitumoral outcomes (Bernstein et al., 2018). However, the ACE2–Ang (1–7)–Mas branch of the RAS serves as a negative regulator of the system that counters ACE–Ang II–AT1R branch activities (Kuba et al., 2013); therefore, as ACEi and ARB were shown to upregulate ACE2 (Kriszta et al., 2021; Parit & Jayavel, 2021), thereby modifying the balance between the canonical and noncanonical axes of the RAS, concerns have been raised over a potential negative impact of ACEi/ARB treatments on immune responses to pathogen infection.

As hypertension is currently recognized as a chronic inflammatory state linked to imbalances of the immune system (Zhang et al., 2022), a number of studies have examined how the most commonly prescribed drugs for hypertension—that is, ACEi and ARB—can affect immune responses. In this context, immune regulation by ACEi and ARB is primarily linked to the prevention of inflammatory cytokine production, the reduction of adhesion molecule expression, and curtailment of plasma C‐reactive protein (Bryniarski et al., 2022).

Studies aimed at clarifying the advantages/disadvantages of RAS‐blockade on immunity have produced conflicting results. Thus, in healthy individuals, 1‐week ACEi (ramipril) administration reduced the bactericidal action exerted by isolated neutrophils, as well as neutrophil ROS production (Cao et al., 2021). These results are in agreement with observations in mice where in vivo ACEi (ramipril and lisinopril)—but not ARB (losartan)—treatments impaired bacterial killing by neutrophils (Cao et al., 2021). Findings in a model of mouse chronic viral myocarditis provoked by coxsackievirus B3, indicate that losartan treatment can reduce mortality, necrosis, and inflammation by upregulating Th2 responses (as indicated by higher IL‐4 serum levels compared to untreated virus‐infected mice), and downregulating Th1 and Th17 responses (as suggested by the reduction of IFN‐γ/TNF‐α and IL‐17 serum levels, respectively, vs. untreated virus‐infected mice), without reducing viral titers (Zhang et al., 2013).

Of note, as already mentioned above, ACEi and ARB can upregulate ACE2 (Kriszta et al., 2021; Parit & Jayavel, 2021), which generates Ang (1–7). Interestingly, work with RAW 264.7 macrophages showed that Ang (1–7) induces monocyte/macrophage migration mediated by Mas receptor activation; in addition, intrapleural Ang (1–7) injection induced leukocyte infiltration into the pleural cavity of mice, and when Ang (1–7) was injected into mouse intra‐articular cavity, it prompted leukocyte migration into the knee joint. Furthermore, in a model of self‐resolving *E. Coli* peritonitis, the Ang (1–7)/MasR axis stimulated macrophage recruitment, CCL2 production (a cytokine involved in the recruitment of monocytes, memory T cells, and dendritic cells to inflammation sites that result from tissue injury or infection), and phagocytosis of bacteria (Zaidan et al., 2022). Moreover, in the course of experimental *Mycoplasma pneumoniae* infection in mice, oropharyngeal Ang (1–7) administration was associated with the reduction of both airway inflammation and bacterial burden; the same authors reported that addition of Ang (1–7) to RAW 264.7 macrophages dose dependently curbed TNF‐α generation, whereas it boosted *Mycoplasma pneumoniae* elimination (Collins et al., 2021).

Altogether, the above evidence indicates that both ACEi and ARB—by stimulating ACE2 upregulation/Ang (1–7) production—may contribute to pathogen clearance, although this issue is not completely settled.

Regarding the advantages or disadvantages of ACEi/ARB administration on immune responses, sizable results do not support the concept that ACEi treatment is associated with a higher risk of adverse immune outcomes compared with that of ARB (Oosthuizen & Sturrock, 2022).

### 2.3 Changes in the activity and responsiveness of RAS during aging

The activity and responsiveness of the RAS are modified during normal aging. Thus, in older animals and humans, circulating renin, aldosterone, and Ang II activities are reduced, which is instrumental to the higher incidence of electrolyte and fluid disarrangements in this age group compared with younger individuals (Anderson, 1997; Arnold et al., 2013). However, there is a dissociation between the behaviors of the circulating RAS and tissue RAS in aging, as revealed by the excessive responses to tissue Ang II that occur in aged animals as a result of increased tissular expression of AT1R and Ang II generation (Carey, 2007; Wang et al., 2003; Yoon et al., 2016).

Furthermore, with reference to the protective axis of the RAS, the circulating Ang (1–7) content is diminished in aging humans relative to younger subjects (Vargas‐Castillo et al., 2020), Mas receptors and ACE2 are reduced in the aorta of aged mice (Yoon et al., 2016), and aortic vasodilation in response to Ang (1–7) is curtailed in aged female mice (Costa‐Fraga et al., 2018).

Regarding circulating Ang (1–7) levels in humans, different researchers found dissimilar plasma contents (Table 1). The table shows that the reported values for human plasma Ang (1–7) are either in the ones, tens, hundreds, or thousands of pg/mL. It is feasible that these differences arise from the dissimilar techniques and/or disparate sample processing used.

**TABLE 1:** Human plasma Ang (1–7) values reported by different authors.

Reference	Patients	Technique	Values
Vargas‐Castillo et al. (2020)	Aging and overweight/obese subjects	Capillary zone electrophoresis with UV detection by photodiode array	20–40 years: ≈0.6 pmol/mL (539.4 pg/mL) 40–60 years: ≈0.4 pmol/mL (359.6 pg/mL)
Hisatake et al. (2020)	Acute heart failure patients versus healthy controls	ELISA	Acute heart failure. 2.4 ng/mL (2000.4 pg/mL) Healthy controls: 3.1 ng/mL (3000.1 pg/mL)
Velloso et al. (2007)	Preeclamptic and normotense pregnant women	RIA	Preeclamptic: 16.9 ± 1.2 pg/mL Normotense pregnant: 21.6 ± 1.1 pg/mL
Sullivan et al. (2015)	Healthy women and men	ELISA	Women (Avg 26 year): 49.5 ± 4.4 fmol/mL (44.5 pg/mL) Men (Avg 26 year)::35.9 ± 3.1 fmol/mL (32.3 pg/mL)
Chappell (2016)	Review of different studies: healthy subjects and hypertensive patients	RIA; immunoreactivity (antiserum against Ang‐(1–7) raised by the authors)	5–80 fmol/mL (4.49–71.9 pg/mL)

*Note*: The determination of human plasma Ang (1–7) contents has generated divergent results among different researchers. These contradictory results might arise from the contrasting techniques and sample processing employed. Ang‐(1–7) molecular weight = 899.02 g/mol.

Previous studies found that the AT1R/Mas receptor ratio was higher in renal nuclei obtained from older sheep, which may be responsible for the higher generation of ROS in response to Ang II/AT1R that was observed in the older animals (Gwathmey et al., 2010). As a whole, these observations acquire special relevance when taking into account that Ang (1–7)—by acting on its Mas receptor—protected human umbilical vein endothelial cell (HUVEC) cultures from Ang II‐induced senescence; Ang (1–7)'s effect was mediated by activation of klotho followed by activation of the cytoprotective Nrf2/heme oxygenase‐1 (HO‐1) pathway (Romero et al., 2019).

Interestingly, the distinctive early aging phenotype associated with ACE2 deficiency (ACE2 KO mice) was not observed in Ang (1–7) receptor deficient mice (Mas KO mice), which suggests that the antiaging actions of ACE2 are Mas independent. However, administration of Ang (1–7) to Mas KO mice did not mitigate age‐related muscle weakness, although it improved muscle strength in both aged wild‐type and ACE2 KO mice (Takeshita et al., 2023). These observations indicate that Ang (1–7)'s antiaging effects are exerted through the Mas receptor. Based on present knowledge, the RAS‐independent mechanisms of ACE2's antiaging actions were discussed in Takeshita et al. (2023).

In the context of the abovementioned results, age‐related conditions, such as myocardial infarction, atherosclerosis, hypertension, and diabetes are associated with RAS over‐activation (Yi et al., 2022).

In brief, it is feasible that the simultaneity of elevated injury promoted by tissue Ang II activity together with reduced Ang (1–7) protective signaling participates in the induction of tissue and organ damage in aging.

### 2.4 RAS contribution to age‐related decline

Accumulating evidence indicates that the RAS contributes to the aging process across species. Thus, genetic or pharmacological RAS manipulation increases lifespan in *Caenorhabditis elegans*, *Drosophila*, and rodents [reviewed in Hisatake et al. (2020)]. In humans, exceptional longevity is associated with certain ACE gene alleles (Egan et al., 2022) that correspond to ACE polymorphism rs4340. This polymorphism consists of either the presence or absence of a 287 base pair (bp) Alu repetitive element in intron 16 of the ACE gene. Alu elements are mobile DNA fragments that are present as repetitive sequences within a genome. Alu element's name originates from the fact that they contain a recognition site for the restriction endonuclease known as AluI. Alu mobile elements are associated with health conditions as a result of insertional mutagenesis—that is, they can disrupt gene function when inserted within a gene—also, since they are present as substantially repetitive sequences, they can cause genetic deletions and duplications through nonallelic homologous recombination [for additional information, please see Ade et al. (2013)].

The ACE variant characterized by the presence of the 287 bp Alu repeat is known as I (insertion) allele, whereas that one distinguished by its absence is recognized as D (deletion) allele. Regarding the consequences of the ACE I/D polymorphism, a study using healthy individuals found that this polymorphism accounted for ≈50% of the variance of serum ACE contents, and in subjects with the D/D ACE genotype serum ACE immunoreactivity was 25% and 65% higher than in those holding I/D and I/I genotypes, respectively (Rigat et al., 1990). Later studies confirmed that in healthy subjects the D/D genotype is associated with both higher plasma/serum ACE activity (Agerholm‐Larsen et al., 1999; Tiret et al., 1992) and cardiac ACE activity (Danser et al., 1995) when compared with the I/D and I/I genotypes.

Of note, two meta‐analysis (Garatachea et al., 2013; Revelas et al., 2018) revealed that extreme longevity (centenarians and individuals >85 years old) is positively associated with the presence of the D allele. However, there are conflicting results among a number of studies that addressed the effects of different ACE variants on human life duration. To settle this issue, a very recent work used an artificial intelligence‐assisted meta‐analysis to investigate the contribution of the ACE gene to human longevity (Li & Murakami, 2023) which confirmed that the D/D genotype is positively associated with extreme longevity, whereas the I/I genotype is negatively associated with the latter trait.

Surprisingly, the D allele is positively associated with cardiovascular disease, arteriosclerosis, diabetic microvascular derangements [reviewed in Sayed‐Tabatabaei et al. (2006)], and pneumonia (Nie et al., 2014); whereas there are conflicting data regarding a positive association of the D allele with high blood pressure, and as a whole the results indicate a minute influence of the D allele on hypertension (Sayed‐Tabatabaei et al., 2006), a recent meta‐analysis reported an association between the ACE gene D allele and a higher susceptibility to essential hypertension, a condition recognized to be polygenic, that is, several genes contribute to its pathogenesis (Liu et al., 2021). In this scenario, the D/D genotype and serum ACE activity were significantly higher in myocardial infarction patients compared with healthy controls (Pulla Reddy et al., 2010).

However, in an Indian population, among ST‐elevation myocardial infarction (STEMI) patients, the ACE polymorphism was not associated with ACE activity, and the latter showed ample variation among individuals of the same genotype (Moorthy et al., 2021).

Therefore, there is a disagreement between studies indicating that the D allele is a risk factor for age‐associated diseases that may lead to mortality and the observation of a positive association of the D/D genotype with extreme longevity. It was suggested that the ACE I/D polymorphism may constitute a case of antagonistic pleiotropy (Revelas et al., 2018), by which at young ages the D allele may enhance the risk of disease but at older ages it may provide a survival advantage. It was also proposed that the D allele may impact on longevity as a result of positive actions on tissue repair (Eisenlohr et al., 1992), immune responses (Ehlers & Riordan, 1989), and muscle strength conservation (Revelas et al., 2018). Nevertheless, as the complete function of human ACE is still unknown, it is feasible that other aspects of ACE's role will be described hereafter that may clarify its involvement in longevity (Sayed‐Tabatabaei et al., 2006).

Regarding the effects of ACEi on the different variants of ACE polymorphism rs4340, in the Genetics of Hypertension‐Associated Treatment (GenHAT) Study, Arnett et al. (2005) hypothesized that after 6 months of antihypertensive treatment, the blood pressure lowering response of hypertensive patients carrying the ACE D/D genotype versus those bearing ACE I/D or I/I genotypes, would be superior in lisinopril (an ACEi)‐treated patients compared with an aggregated group of patients treated with other antihypertensive drugs (chlorthalidone, amlodipine, or doxazosin). It should be remembered that ‐as mentioned earlier—those individuals carrying the D/D genotype present higher serum ACE immunoreactivity/activity versus either I/D or I/I genotypes. However, the study results did not sustain the author's hypothesis, as the diminution in systolic blood pressure was inferior in the lisinopril‐treated D/D genotype patients relative to that observed in the I/D or I/I patients. Contrarily, blood pressure lowering in the aggregated “other antihypertensive” group showed no differences among the D/D, I/D, and I/I ACE genotype groups. In conclusion, the GenHAT study did not support the concept that the ACE I/D polymorphism might modify ACEi‐mediated blood pressure lowering when compared with other amply prescribed antihypertensive drugs. In the same study, no differences were found for fatal coronary heart disease, nonfatal myocardial infarction and all‐cause mortality among the different treatment groups. Therefore, the study results oppose the well‐known proposal that the beneficial actions of ACEi in hypertension rely upon the patient's ACE activity, and consequently on the type of ACE I/D genotype.

The evidence discussed in the previous paragraph seem to be consistent with the observation that a generous inter‐individual variability in plasma ACE levels exists in healthy subjects, as ACE levels can vary by a factor of five among individuals (Alhenc‐Gelas et al., 1991).

The beneficial effects of RAS‐blockade in normotensive aging rodents were recently reviewed (Egan et al., 2022), and they variously include the improvements of renal and cardiac health, reductions of tumor incidence, age‐related hypertension, frailty and adiposity, the enhancement of muscle strength, healthier skin, along with work from our laboratory demonstrating that RAS‐blockade protects mitochondrial number (Ferder et al., 1993; Inserra et al., 1995) and function (de Cavanagh et al., 2003; de Cavanagh, Ferder, et al., 2008) from the effects of mouse aging, and increases mice survival (Ferder et al., 1993). According to our understanding, these reports were the first to show that ACEi treatment preserves mitochondria and enhances survival in aging rodents. The amelioration of mice survival was later reproduced in male rats (Ferder et al., 2002). Furthermore, in female mice treated with enalapril from weaning up to 18 or 24 months, the cardiac antioxidant enzyme mitochondrial Mn‐superoxide dismutase (MnSOD), mitochondrial number and cyclin expression were higher, and apoptosis markers were lower than those observed in hearts obtained from untreated animals (Ferder et al., 1998).

Considering these results, we hypothesized that RAS‐blockade interferes with the natural aging mechanisms, and that the RAS contributes to the aging process (Basso et al., 2005).

Next we investigated whether ACEi protected mitochondria by acting as an antioxidant, and showed that in female mouse tissues both enalapril and captopril protected cells from oxidant injury by shifting the oxidant/antioxidant equilibrium toward antioxidants (de Cavanagh et al., 1997, 2000). In accordance with the latter results, the circulating levels of the red blood cell antioxidants glutathione and Se‐glutathione peroxidase, as well as plasma beta‐carotene, were higher in ACEi (enalapril)‐treated hemodialysis patients than in patients that received calcium antagonists (de Cavanagh et al., 1999).

To increase our understanding on the role of RAS in the aging process, we examined whether RAS‐blockers protected mitochondrial function by boosting antioxidant defenses. In male Wistar rats, treatment with enalapril or losartan—administered from 14 months old to 22 months old—mitigated or blocked age‐associated renal mitochondrial dysfunction and ultrastructural changes, which were accompanied with an enhancement of kidney glutathione redox status (de Cavanagh et al., 2003), implying that enalapril and losartan may counteract the signs of aging by reducing oxidative injury to mitochondrial structure/function.

Taking into account that both mitochondrial dysfunction and RAS contribute to tissue damage in hypertension (Ramachandran et al., 2002) and diabetes (Schrauwen & Hesselink, 2004), we investigated whether RAS‐blockade could protect renal mitochondria from damage associated with both diseases. In male spontaneously hypertensive rats (SHR), losartan administration counteracted the alterations in renal mitochondrial membrane potential, mitochondrial H_2_O_2_ production rate, uncoupling protein (UCP)‐2 content, Mn‐SOD, mitochondrial NOS, and cytochrome oxidase activities that were found in untreated SHR (de Cavanagh et al., 2006). In streptozotocin‐induced diabetic male rats, losartan protected kidney mitochondria against changes in membrane potential, H_2_O_2_ production rate, and pyruvate content; however, plasma glucose content continued to be elevated compared with nondiabetic rats (de Cavanagh, Ferder, et al., 2008). In both studies, the Ca2+ channel blocker amlodipine reduced blood pressure to the same degree as losartan but it did not display any beneficial effects on renal mitochondria, which underscores the notion that RAS‐blockade protects against age‐related changes independently from its action on blood pressure. This concept was further illustrated when SHR that had been treated with a non‐antihypertensive dose of enalapril (from 3 months old up to 8 months of age) displayed reduced heart weight and myocardial fibrosis compared with untreated SHR, even though blood pressure remained equivalently high in both groups in relation to the normotensive controls. In the same study, SHR that had been treated with enalapril showed lower matrix metalloprotease‐2 and Mn‐SOD activities, and higher NADH/cytochrome c oxidoreductase, eNOS, and mtNOS activities, as well as higher eNOS protein compared with untreated SHR. Hence, independently from its effect on blood pressure, enalapril preserved heart tissue and mitochondria from damage inflicted by hypertension (Piotrkowski et al., 2009). In this regard, convincing results [reviewed in Saravi et al. (2021)] demonstrate that the influence of RAS‐blockers on tissue RAS—together with circulating RAS‐blockade—provides the basis for the various favorable effects of these drugs.

In agreement with the above findings in the kidney, other investigators showed that blocking Ang II signaling ameliorated cardiac mitochondria energy generation in experimental models of ischemia and post‐infarction remodeling (Monteiro, Duarte, et al., 2005; Monteiro, Gala, et al., 2005; Ochiai et al., 2006).

In addition, we investigated the effects of RAS‐blockade on age‐related mitochondrial DNA (mtDNA) alterations. Enalapril or losartan administration to rats for 16.5 months had no favorable effect on the age‐related accumulation of liver mtDNA “common deletion,” but both drugs attenuated the diminution of mtDNA content (de Cavanagh, Flores, et al., 2008). Also, enalapril and losartan increased nuclear respiratory factor (NRF)‐1 and peroxisome proliferator activator receptor‐gamma coactivator‐1a (PGC‐1a) mRNA liver contents, compared with untreated rats. These results support the observed amelioration of mitochondrial function and lowering of oxidant production associated with RAS‐blockade, inasmuch as mitochondrial respiratory capacity is positively related to both the absolute number of mtDNA molecules (Bentlage & Attardi, 1996; Foote et al., 2018) and increased NRF‐1 and PGC‐1 expression (Finck & Kelly, 2006; Scarpulla, 1997; Wu et al., 1999); moreover, PGC‐1 is a defensive factor against oxidant production and damage (Spiegelman, 2007).

Interestingly, studies in worms, flies, mice, and humans that addressed age‐related changes in gene expression, found that only those genes that code for components of the mitochondrial electron transport system displayed a common trend toward decreased expression. In contrast, most of the other age‐related changes in gene expression are unique to each species [reviewed in Kim (2007)].

Regarding the relevance of restoring mitochondrial function during aging, as pointed out by Inagami (2011), RAS‐blockade may turn out to be a successful treatment option to increase lifespan.

Additional data relative to the contribution of RAS to the aging process was afforded by a study showing that—in agreement with our previous work in which RAS was inhibited by ACEi or ARB treatment—disruption of the AT1R gene enhances mice longevity, diminishes cardiac damage, vascular damage, and oxidative injury in multiple organs; furthermore, it also enhances renal mitochondria number and the expression of nicotinamide phosphoribosyl‐transferase (Nampt, whose protein product boosts mitochondrial NAD+, supplying the co‐substrate for SIRT3) and sirtuin 3 (Sirt3)—two genes that promote survival (Benigni et al., 2009). An additional connection between RAS activation and sirtuins was observed when incubation of murine tubular epithelial cells in the presence of Ang II led to downregulation of Sirt3 mRNA, an effect that was prevented by blockade of AT1R (candesartan). In view of these observations the authors suggested that Ang II/AT1 signaling may be interfered with in order to manipulate mammalian lifespan.

Sirtuins (SIRT 1–7 in humans) are nicotinamide adenine dinucleotide (NAD+)‐dependent deacetylases that modulate the function of their biological targets by eliminating acetyl groups from proteins containing acetyllysine. In response to stress and toxicity, sirtuins enhance organism and tissue survival across animal species (Yang & Sauve, 2006); however, only SIRT3 seems to directly contribute to increased lifespan in humans (Bellizzi et al., 2005). Cellular stress leads to SIRT3 translocation from nuclei to mitochondria (Scher et al., 2007) where this sirtuin protects against mitochondrial ROS (Dikalova et al., 2020; Xiong et al., 2013) by prompting deacetylation—and thereby activation—of the antioxidant enzyme mitochondrial MnSOD (Tao et al., 2014). The key role played by mitochondrial ROS in promoting vascular damage and senescence was demonstrated when Ang II was infused to Sirt3‐deficient mice and Sirt3 transgenic mice. In Sirt3‐deficient mice, Sirt3 depletion was associated with hypertension and hyperacetylation of MnSOD, conducting to the loss of MnSOD activity and to mitochondrial oxidative stress, that were accompanied with vascular damage and early vascular senescence. Contrarily, mice globally overexpressing Sirt3 (Sirt3OX) were protected from Ang II‐induced vascular oxidative stress and damage, and showed an alleviation of hypertension (Dikalova et al., 2020).

In addition to the favorable mitochondrial actions offered by RAS‐blockade during aging, as described in the present article, a thorough list of the beneficial mitochondrial effects afforded by ACEi or ARB, with a focus on cardiovascular disease, can be found in a recent article (Betiu et al., 2022).

Of note, in clinical studies, ACEi were shown to ameliorate bone metabolism or to reduce the risk of bone fractures (Lynn et al., 2006; Perez‐Castrillon et al., 2003; Rejnmark et al., 2006). Likewise, the ARB olmesartan, abated the manifestations of osteoporosis in ovariectomized SHR, whereas hydralazine had no effect (Shimizu et al., 2008), and in osteoporotic rats telmisartan enhanced bone mineral density (Mahmoud et al., 2022).

When comparing the effects of different antihypertensive agents on the risk of dementia and cognitive decline in older adults (Liampas et al., 2022), those patients treated with ARBs were better protected against the deterioration of cognitive functions in comparison with other antihypertensive therapies, and—seemingly—this action was not related to an effect on blood vessels. Human studies (Gouveia et al., 2022) on the beneficial effects of ACEi or ARB in slowing down cognitive decline, have disclosed that those RAS‐blockers that can get through the blood–brain barrier outperform those that cannot cross it.

The above evidence indicates that RAS‐blockers can target the brain RAS, suggesting that they are a promising intervention against Alzheimer's disease.

With regard to the influence of ACEi/ARB on human mortality, a recent review (Kulkarni et al., 2022) listed diverse observational and interventional trials, of which—according to our own analysis—roughly 70% demonstrate an association between RAS‐blockade and reduced mortality.

In a very recent study (Strong et al., 2022) conducted at three different test sites, captopril (an ACEi) treatment—started at 5 months of age—enhanced median lifespan in male mice by 13% (*p* = 0.001) and by 6% (*p* = 0.002) in female mice, according to the combined results of the three sites. However, the interpretation was complex by the curiously low survival of female and male controls at one of the sites. To overcome this complication, the authors provided an additional two‐site analysis that excluded the site where the low survival of controls had occurred. The latter analysis showed that captopril increased female lifespan by 4% (*p* = 0.04), although in males a 6% (*p* = 0.26) increase was observed. When the data were analyzed individually by site, captopril significantly increased female median lifespan at two of the test sites (by 10% and 7%, respectively), and had no effect at the third site; whereas in males captopril enhanced median lifespan only at one test site (27%, *p* < 0.001). In this scenario, the authors suggested that further studies using several doses of captopril, will probably produce fruitful results.

As a whole, the above data indicate that in aging rodents and humans the benefits offered by RAS‐blockade include the lengthening of both health‐ and lifespan.

In this context, it should be noted that current evidence demonstrates that vertebrate species longevity correlates exclusively with the production rate of ROS by mitochondria, and with the extent of membrane fatty acid unsaturation in tissues (Barja, 2014). The influence of the two latter parameters on longevity is consistent with the mitochondrial free radical theory of aging (Harman, 1972; Miquel et al., 1980) which proposes that maximal lifespan in mammalians is negatively related with both the occurrence of oxidative damage and membrane responsiveness to oxidation.

### 2.5 Mitochondria‐derived peptides (MDPs), aging, and RAS

In humans, the circular mtDNA encodes for 13 protein subunits of the oxidative phosphorylation complexes, 2 rRNAs, and 22 tRNAs (Barshad et al., 2018). However, regulation of the mitochondrial genome has a higher level of complexity, in part due to the existence of small open reading frames (sORFs) that generate various microproteins known as mitochondria‐derived peptides (MDPs) (Miller et al., 2022). At present, eight MDPs have been reported: humanin, MOTS‐c (mitochondrial open‐reading frame of the 12S ribosomal RNA type‐c), and SHLPs 1–6 (small humanin‐like peptides 1–6).

Concerning the role of MDPs in aging, age‐associated conditions—including dysfunction of coronary endothelium, type 2 diabetes, and Alzheimer's disease—have been linked to reduced MOTS‐c or humanin plasma contents (Du et al., 2018; Muzumdar et al., 2009; Qin et al., 2018; Ramanjaneya et al., 2019; Widmer et al., 2013; Yen et al., 2018; Zarate et al., 2019). Interestingly, circulating plasma humanin (Muzumdar et al., 2009) and MOTS‐c (D'Souza et al., 2020) levels diminish with aging in mice and humans. Furthermore, in *Caernorhabditis elegans* overexpression of humanin increased lifespan (Yen et al., 2020), and humanin injected twice a week during 14 months to female mice of middle age promoted healthspan, but did not enhance lifespan probably due to the short (≈20 min) half‐life of humanin (Yen et al., 2018).

Although in humans circulating MOTS‐c decrease with age, in old and middle‐aged healthy men skeletal muscle MOTS‐c expression was found to be approximately 1.5 times higher than in young subjects, and it was associated with slow‐type muscle fibers and augmented muscle function in older subjects (D'Souza et al., 2020).

Of note, under conditions of metabolic stress (such as oxidative stress or glucose restriction) MOTS‐c translocates from mitochondria to the cell nucleus where it directly regulates the expression of genes involved in adaptive responses to favor homeostasis (Kim et al., 2018).

Regarding the relation between MDPs and RAS, in a model of vascular calcification induced by vitamin D3 and nicotine in the rat, daily intraperitoneal administration of MOTS‐c for 4 weeks significantly reduced blood pressure and aortic stiffness, and ameliorated echocardiographic parameters through activation of the AMPK pathway and reduction of AT1R and ET‐B receptors (Wei et al., 2020). Since, as already mentioned, aging is associated with lowering of circulating MOTS‐c, it can be hypothesized that low MOTS‐c levels will be accompanied with increased AT1R expression and signaling; therefore, ACEi or ARB treatments may attenuate/prevent the deleterious actions of RAS in aging by neutralizing at least some of the effects of low MOTS‐c contents.

In conclusion, although the available data are still scarce, it is possible to speculate that ACEi and ARB may protect from age‐associated decay by modulating MDPs effects.

## 3 EPIGENETICS AND AGING

The functional decline that defines aging is accompanied with epigenetic changes in genome expression (Wang et al., 2022). Epigenetics refers to the reversible regulation of gene activity by modification of the structure of DNA and/or its accompanying proteins (primarily, histones) without alteration of DNA sequence, and also by posttranscriptional modifications mediated by noncoding RNA and RNA modification (Bird, 2007). DNA and protein modifications include DNA methylation, and histone acetylation, methylation, ubiquitination, phosphorylation, and sumoylation. These epigenetic changes were found to participate in the promotion of aging‐associated conditions. Ongoing research in this field is helping to delineate a modern theory of aging that includes epigenetics as a mechanism that underlies age‐related changes (Li et al., 2022).

It should be noted that the reversibility of epigenetic alterations offers the possibility to search for interventions to interfere with the aging process. Calorie restriction (CR) increases healthspan and longevity in animal species, as well as in humans (Dorling et al., 2020). The beneficial effects of CR in aging are—at least in part—mediated by epigenetic changes (Gensous et al., 2019). In a previous publication (de Cavanagh et al., 2011), we discussed the converging effects of CR and RAS‐blockade during aging, and also how these overlapping protective actions involve alterations in mitochondrial function.

In this scenario, the beneficial actions displayed by RAS‐blockade in aging might be mediated by epigenetic changes and the ensuing modification of gene expression and translation rates, which could lead to the reduction of mitochondrial ROS production and oxidative damage. Various studies support the contribution of Ang II to epigenetic alterations. Thus, increased expression of aortic Na + ‐K + ‐2Cl‐cotransporter 1 mediated by epigenetic histone changes was observed in rats made hypertensive by Ang II infusion (Cho et al., 2012). Also, some of the epigenetic modifications associated with diabetic nephropathy in type 2 diabetic db/db mice were reversed by losartan treatment, pointing to the involvement of AT1R in the induction of chromatin changes that affect the expression of harmful genes (Reddy et al., 2014). In addition, in cultured AC16 cardiomyocytes, Ang II prompted DNA methylation of the MIR150HG gene, thereby reducing the expression of miR‐150‐5p microRNA which enhanced AT1R expression (Qian et al., 2019). Moreover, in cultured atrial cardiomyocytes, Ang II stimulated nuclear export of histone deacetylase 4 and 5 (HDAC4 and HDAC5) into the cytoplasm—which inhibits chromatin condensation—and allowed the epigenetic transcription of hypertrophy‐related genes. These Ang II‐related pro‐hypertrophic actions were partly inhibited by losartan treatment (Zheng et al., 2022). The two last studies were conducted on cultured cells indicating that Ang II can induce epigenetic changes independently of is effect on blood pressure.

Finally, how RAS components can also be targets of epigenomic modifications has been discussed elsewhere (Wise & Charchar, 2016).

At present, there are no studies indicating whether RAS peptides can influence epigenetic modifications by acting on the nuclear AT1R or Mas receptor, as opposed to the cell membrane receptors.

Summarizing, based on the above results, it is feasible that RAS‐blockade may interfere with the aging process by modifying gene expression through epigenetic changes.

## 4 SIGNALING PATHWAYS RECOGNIZED TO PARTICIPATE IN AGING

The discovery of gerontogenes, that is, genes that are involved in conserved pathways across species and enhance longevity when they are mutated or overexpressed, led to the search for therapeutic compounds able to interfere with the aging process in humans. As aging is associated with the evolving deterioration of homeodynamics [which refers to the dynamic capacity of living organisms to respond through diverse pathways to internal or environmental stress by neutralizing any disruption that endangers their survival (as opposed to homeostasis which is a special case of homeodynamics, but prioritizes the stability of the internal milieu in the face of disruption) (Lloyd et al., 2001)], the most probable gerontogene candidates are those genes that participate in homeodynamic pathways (Rattan, 2004). The concept of homeodynamic space (or buffering capacity) refers to all the pathways, genes, and gene products involved in maintenance, repair, and defense systems that counteract damaging events and ensure survival and health preservation. A successful homeodynamic space comprises three main features: stress response, damage control, and constant tissue remodeling, and is responsible for the strength and ability of biological systems to recover from adversity, and, in the end, survival capability (Rattan, 2020).

The signaling pathways that were found to participate in the regulation of aging include TOR, AMPK, insulin/IGF‐1, TGF‐beta, NF‐kB, PI3K, MAPK, PKC, Notch, and Wnt (Moskalev et al., 2014).

From invertebrates to mammals, when the environmental setting is advantageous these signaling pathways regulate organismal energy equilibrium, cell's ability to change identity, and the systems that promote homeostasis, growth, and reproduction (Barzilai et al., 2012). However, when conditions become rough, hormonal growth promotion is obstructed, and proteins that offer stress resistance are activated (Kim, 2007).

In this section we discuss the involvement of the latter signaling pathways in aging, their relations with mitochondria—as deterioration of mitochondrial function is one of the distinctive traits of aging—and how the RAS modifies their signaling functions.

In a previous review (de Cavanagh et al., 2015) we examined how RAS‐blockade downregulates mechanistic target of rapamycin (mTOR) and growth hormone/IGF‐1 signaling, and also stimulates AMPK activity (together with the upregulation of klotho, sirtuin, and vitamin D receptor expression), and proposed that at least some of the benefits of RAS‐blockade in aging are mediated through regulation of these intermediaries and the associated signaling to mitochondria. In the present article, we expand the information on the impact of RAS‐blockade on other signaling pathways that are known to participate in the regulation of aging, that is, TGF‐beta, NF‐kB, PI3K, MAPK, PKC, Notch, and Wnt.

### 4.1 mTOR

mTOR is a nutrient sensing protein kinase that modulates eukaryotic cell growth and metabolism. It forms two structurally and functionally different kinase complexes: mTORC1 and mTORC2. mTOR activation variously impacts on mitochondrial function (Scarpulla, 1997) (see below).

From yeast to mice, mTOR signaling was shown to modulate the aging process, pointing to mTOR hyperactivation as a relevant event in the promotion of aging (Stallone et al., 2019).

Available evidence demonstrates that pharmacological mTOR inhibition extends lifespan and provides protection against age‐related conditions in preclinical models (Papadopoli et al., 2019). Several mTOR inhibitors—such as rapamycin, tacrolimus, everolimus—are approved for clinical use in the context of various indications; however, the occurrence of adverse effects prevents their administration to healthy individuals.

#### 4.1.1 mTOR and mitochondria

mTOR regulates abundant features of mitochondrial function, including mitochondrial apoptosis and biogenesis, mitohormesis (a process involving the production of a reduced amount of mitochondrial ROS that act as signaling agents that lead to a protective response to an injurious stimulus), mitochondrial unfolded protein response, mitochondrial autophagy, and retrograde signaling [reviewed in Wei et al. (2015)].

mTOR signaling is closely related to AMP‐activated protein kinase (AMPK); in fact, the combination of mTOR and AMPK signaling regulates several pathways involved in mitochondrial dysfunction, oxidative stress, and the preservation of immune responses (Cetrullo et al., 2015; Maiese, 2020).

#### 4.1.2 mTOR and RAS

In a previous article (de Cavanagh et al., 2015), we summarized the results of various studies indicating that RAS‐promotion of tissue injury is mediated, at least partly, by inducing mTOR activation, and both tissue damage and mTOR signaling are mitigated/forestalled by blocking different RAS components. This knowledge led us to propose that mTOR regulation is one of the mechanisms involved in the beneficial actions of RAS‐blockade in aging.

### 4.2 IGF‐1

Abundant evidence has connected the insulin/insulin‐like growth factor (IGF‐1) signaling pathway with the aging process (Barzilai et al., 2012; Kenyon, 2010). Growth hormone (GH)‐mediated activation of GH receptors induces the synthesis and secretion of IGF‐1. In humans, aging is associated with definite declines in GH secretion and circulating IGF‐1 levels (Junnila et al., 2013).

Trying to separate the repercussions of the concurrent decline of GH and IGF‐1 levels is difficult for the reason that not all GH's effects are mediated by IGF‐1. In addition, GH and IGF‐1 can exert contrasting or even antagonistic actions on certain events or target tissues (Junnila et al., 2013), and in some organs—such as the liver—IGF‐1 expression is dependent on GH, while in others it is not (Lupu et al., 2001). As a result of the above complexity, studies conducted in humans have produced contradictory results and indicated that age‐related changes in the levels of IGF‐1 are a double‐edged sword (Gubbi et al., 2018; Poudel et al., 2020).

#### 4.2.1 IGF‐1 and mitochondria

Some of the work addressing the relation between IGF‐1 signaling and mitochondria was conducted on cancer cell lines due to previous reports suggesting that reductions of insulin and IGF‐1 pathway signaling might turn out to be beneficial in cancer prevention and therapy (Pollak, 2008). In cultured MCF‐7 (a human breast cancer cell line) and HeLa cells (a human cervical cancer cell line), IGF‐1 signaling was shown to promote the expression of pyrimidine nucleotide carrier 1 (PNC1), a protein that carries deoxynucleotides, that is, the precursors for mtDNA synthesis, from the cytoplasm into mitochondria; in turn, PNC1 was observed to regulate the duplication of mtDNA and its transcription, mitochondrial ROS, and energy production, as well as the capacity of cancer cells to acquire invasive characteristics (Favre et al., 2010).

Huntington's disease—an inherited neurodegenerative condition caused by mutation of huntingtin protein—is associated with dysfunctional mitochondria that display a deficiency in energy production. In lymphoblasts collected from patients with Huntington's disease, IGF‐1 treatment enhanced mitochondrial membrane potential, ATP levels, oxygen consumption, and cytochrome c content (Naia et al., 2015).

On account of ample evidence pointing to the association of mitochondrial dysfunction with various diseases, as well as to the relevant role played by IGF‐1 in the protection of mitochondria, the administration of low IGF‐1 doses was proposed as a potential treatment aimed at reestablishing mitochondrial function (Sadaba et al., 2016).

#### 4.2.2 IGF‐1 and RAS

Ang II is known to reduce circulating IGF‐1 levels (Brink et al., 1996) but to stimulate tissular expression of IGF‐1.

Available data indicate that cardiac instead of circulating IGF‐1 levels participate in the promotion of renovascular hypertensive left ventricular hypertrophy (LVH). Accordingly, in a model of hypertension and LVH in Goldblatt rats that were treated with either enalapril, losartan, propranolol, or alpha‐methyldopa, left ventricle IGF‐1 levels and left ventricle mass were significantly reduced versus untreated animals. Serum IGF‐1 contents were lower in Goldblatt rats treated with either enalapril, propranolol or alpha‐methyldopa compared with untreated animals, but not in those treated with losartan. These results suggest that local tissue IGF‐1, in lieu of circulating IGF‐1, contributes to the development of pressure overload‐induced LVH in the rat (Jalil et al., 1999).

As a result of the above, it can be speculated that the beneficial effects of RAS‐blockade against age‐related decay are mediated—at least in part—through inhibition of tissular IGF‐1 signaling.

### 4.3 TGF‐beta

Transforming growth factor beta (TGF‐b) are a group of secreted cytokines that regulate cell differentiation, migration, proliferation, adhesion, and apoptosis in a variety of cell types, and in consequence they participate in the control of embryogenesis, tissue homeostasis, and tissue repair (Clark & Coker, 1998). After binding to their plasma membrane receptors, TGF‐b also exert potent profibrotic (Noble & Border, 1997) actions. Derangements in TGF‐b signaling are involved in the development of several human diseases, such as autoimmune conditions, cancer, and fibrosis. Concerning aging and degenerative diseases, TGF‐b was found to regulate mitochondrial energy production and cell responses to oxidative stress (Casalena et al., 2012).

#### 4.3.1 TGF‐b and mitochondria

As mentioned above, TGF‐b is known to regulate mitochondrial energy production.

In cultured human normal bronchial epithelium cells (BEAS‐2B cells), TGF‐b1 enhanced mitochondrial ROS generation, induced the loss of mitochondrial membrane potential, and mitochondrial fission (Patel et al., 2015). Inside cells, mitochondria form an adaptable network of organelles that has the ability to reorganize in response to cellular energy needs. Mitochondrial network organization is tightly regulated, and results from the balance between mitochondrial fusion and fission. Dysregulation of the mitochondrial network is associated to disease, such as hereditary neurodegeneration (Olichon et al., 2007; Verhoeven et al., 2006).

Additional evidence on the relation of TGF‐b and mitochondria was previously reviewed (Casalena et al., 2012).

Recently, in cultured ovine pulmonary arterial endothelial cells, TGF‐b was found to enhance mitochondrial ROS production, depolarize mitochondria, and diminish ATP generation, all of which were mediated by disturbance of carnitine homeostasis (Sun et al., 2020).

Carnitine is a compound that participates in long‐chain fatty acid transport from the cytoplasm to mitochondria to enter beta‐oxidation for the generation of energy.

#### 4.3.2 TGF‐b and RAS

Ang II binding to its AT1R is a key mediator of myocardial and renal fibrosis ‐at least in part‐ by upregulating the expression of TGF‐b (Sun et al., 1998) and/or the TGF‐b receptor known as endoglin (Chen et al., 2004). In both animal models of diabetes and diabetic patients (Brenner et al., 2001), the administration of ACEi or ARB was found to protect from renal decay. In this scenario, RAS‐blockade reduced TGF‐b expression and renal fibrosis (Noble & Border, 1997). Similar results were found in our studies in different animal models—that is, tubulointerstitial lesions induced by experimental hyperoxaluria, kidney disease associated with streptozotocin‐induced type 1 diabetes, aging kidney and myocardium, and fibrotic reaction around mammary implants—in which the amount of TGF‐b in different tissues was significantly lower in RAS‐blocked animals compared with untreated controls (de Cavanagh et al., 2009; Toblli et al., 1999, 2004; Zimman et al., 2007).

Of note, aging and age‐related diseases are associated with perturbations of TGF‐b signaling (Krieglstein et al., 2012; Tominaga & Suzuki, 2019), therefore the favorable effects offered by RAS‐blockade during aging might result—albeit partially—from downregulation of TGF‐b production.

### 4.4 NF‐kB

The ubiquitous transcription factor known as nuclear factor‐kappa B (NF‐kB)—that in mammals consists of a five‐member protein family—regulates the expression of various genes involved in inflammation and immune responses, including those that encode chemokines, pro‐inflammatory cytokines, adhesion molecules, and inflammatory enzymes (Barnes, 1997), as well as genes that regulate cell cycle progression, apoptosis, and cell senescence. In addition, NF‐κB contributes to synaptic plasticity and memory. Interestingly, plenty of studies showed that NF‐kB is the nerve center of a network of intersecting signaling pathways, and therefore is in charge of intricate organismal signaling (Kaltschmidt & Kaltschmidt, 2009; Karin, 2009).

As such, faulty regulation of NF‐κB is involved in inflammatory and autoimmune conditions, cancer, incorrect immune responses, viral infection, and septic shock. The activity of NF‐kB undergoes impressive changes in the course of human and animal aging, with either increased or decreased NF‐kB activity depending on the specific tissue (Giardina & Hubbard, 2002). In fact, NF‐kB is the transcription factor most strongly associated with the aging process. Increases in the expression of NF‐kB are related to a variety of age‐associated degenerative diseases, such as diabetes, osteoporosis, and Alzheimer's disease. Site‐specific inhibition of NF‐kB activity in the skin of aged mice reversed the age‐related changes and gene expression pattern to those observed in younger animals (Adler et al., 2007).

#### 4.4.1 NF‐kB and mitochondria

Work conducted on cultured mouse embryonic fibroblasts showed that the non‐classic NF‐kB signaling pathway is involved in the regulation of mitochondrial morphology, whereas the canonical NF‐kB signaling pathway is not (Laforge et al., 2016).

How NF‐kB impacts on mitochondrial function—by acting from the inside as well as from the outside of the organelle—has been recently reviewed (Albensi, 2019) and is summarized further on in the present and the following paragraphs. Thus, NF‐kB was reported to be present both in the mitochondrial intermembrane space and the inner mitochondrial matrix; also, the p50 and p65 subunits of NF‐kB were observed to be bound to mtDNA, indicating that NF‐kB can regulate the expression of mitochondrial mRNA. Concerning the involvement of NF‐kB in mitochondrial fusion and fission, in cardiac myocytes NF‐kB participates in the promotion of mitochondrial fusion induced by activation of TNF‐alpha receptor 2. It should be remembered that mitochondria form a dynamic network of organelles that can be restructured by fusion and fission to efficiently respond to cell energy requirements.

The two most important functions of mitochondria are the generation of energy (ATP) and regulation of apoptosis. Mitochondria harmonize signals coming from different sources that result in either the activation or inhibition of apoptosis; one of those signals is NF‐kB, whose activation leads to inhibition of apoptosis in most cells. Also, NF‐kB regulates energy production by controlling the balance between cytosolic glucose oxidation (glycolysis) and the mitochondrial electron transport chain; this metabolic adaptation occurs in cancerous and non‐cancerous cells. Furthermore, NF‐kB signaling seems to positively or negatively affect the expression of nuclear genes that code for mitochondrial subunits of the electron transport chain, and also the expression of respiratory complex subunits encoded by mtDNA. Therefore, it was suggested that NF‐kB can alter the activity of enzymes involved in mitochondrial respiration.

Abundant studies indicate that in many age‐associated degenerative diseases—including Alzheimer's—deranged mitochondrial function is a key event that leads to changes in brain metabolism. The effects of amyloid‐beta—a peptide found in the plaques observed in the brains of Alzheimer's disease patients—were studied in the HT22 hippocampal neuronal cell line and in mitochondria isolated from these cells; the cells and mitochondria were incubated in the presence or absence of (a) exogenous Aβ 1–42 (human amyloid beta peptide) and (b) an NF‐kB inhibitor (BAY11‐7082), and mitochondrial function was evaluated. In this study, amyloid beta promoted mitochondrial dysfunction, and this was abolished by the NF‐kB inhibitor pointing to the participation of NF‐kB signaling in amyloid‐induced mitochondrial dysfunction (Shi et al., 2014).

#### 4.4.2 NF‐kB and RAS

Activation of NF‐kB is a key contributor to Ang II‐mediated inflammation (Wolf et al., 2002; Zhang et al., 2005). However, Ang II activates NF‐kB after binding to AT1 and AT2 receptors; accordingly, in an unilateral ureteral obstruction model in mice, treatment with AT1 or AT2 antagonists only partly reduced NF‐kB activation, indicating that dual AT1 and AT2 blockade is needed to thoroughly inhibit inflammation in this model (Esteban et al., 2004).

Additionally, in a model of tubulointerstitial inflammation induced by experimental hyperoxaluria in our lab, both benazepril or losartan treatments significantly reduced tissular NF‐kB immunostaining, as well as various inflammatory markers (IL‐6, MCP‐1, RANTES, and ED1 monocytes/macrophages) (Toblli et al., 2005).

Also, in rat thoracic aorta smooth muscle cells, the induction of IGF‐1 receptor expression by Ang II is mediated by binding of NF‐kB to NF‐kB sites in the IGF‐1 receptor gene promoter (Ma et al., 2006).

In cultured mouse inner medullary collecting duct cells (mIMCD‐K2 cells), treatment with high NaCl induced apoptosis and increased the protein expression of (Pro)renin receptor (PRR) and phosphorylated NF‐kB p65 protein. Inhibition of NF‐kB attenuated the upregulation of PRR, which shows that exposure to high salt promotes PRR expression by activating NF‐kB, thereby contributing to apoptosis (Su et al., 2017).

Thus, the ample evidence supporting the connections among NF‐kB and inflammation, senescence, age‐associated disease, and alteration of mitochondrial function points to RAS‐blockade's interference with NF‐kB signaling as an instrumental factor for ACEi or ARB protective effects against age‐associated changes.

### 4.5 PI3K

Phosphoinositide 3‐kinases (PI3Ks) consist of a family of enzymes (classes I, II, and III PI3K) that participate in cell differentiation, motility, growth, proliferation, survival, and intracellular transport pathways. Several PI3Ks can activate the PI3K/AKT/mTOR pathway, an intracellular signaling pathway directly related to cancer and longevity. In addition, PI3Ks are important components of the insulin signaling pathway. PI3K activation is prompted by various oncogenes and growth factor receptors, and high PI3K signaling is associated with cancer (Fruman et al., 2017). Also, the derangement of microglial PI3K signaling seems to be associated with Alzheimer's disease (Chu et al., 2021).

Notably, in old mutant mice the cardiac suppression of PI3K expression was shown to increase survival, preserve cardiac function, attenuate the expression of senescence markers and the accumulation of lipofuscin in heart tissue, as compared with old non mutant mice (Inuzuka et al., 2009).

#### 4.5.1 PI3K and mitochondria

In organotypic cultures of glioblastoma cells obtained from patients, treatment with a PI3K inhibitor (PX866) stimulated tumor cell motility. PX866 also induced intense changes in mitochondrial morphology and organelle allocation to the cortical cytoskeleton of glioblastoma cells, as well as increased ROS generation when compared with untreated tumor cells (Caino et al., 2015). These results indicate that PI3K signaling can impact on mitochondrial functioning.

In times of nutrient scarcity cells can obtain energy from the autophagy‐mediated degradation of macromolecules in lysosomes, and through lipid catabolism in the mitochondria. In this context, the lysosomal and mitochondrial actions need to be harmonized to ensure a successful outcome. Class III PI3K [composed of a Vps34 catalytic subunit and a Vps15 regulatory subunit (Baskaran et al., 2014)] is a crucial player in the coordination of the different steps that define autophagy (Backer, 2016). However, until recently the impact of class III PI3K on mitochondrial function needed further explanation. Studies aimed at clarifying the latter topic were conducted in mice with liver‐specific Vps15 knockout (KO). They showed that class III PI3K activation contributes not only to the transcriptional induction of autophagy, but also to the transcription of genes related to mitochondrial biogenesis and lipid catabolism (Iershov et al., 2019).

In addition, in rats with lipopolysaccharide (LPS)‐induced acute lung injury, as well as in macrophages with LPS‐induced oxidative damage, the PI3K/Akt pathway was found to promote the activation of heme oxygenase‐1 (HO‐1) which enhanced mitochondrial membrane potential, maintained mitochondrial dynamics (the balance between mitochondrial fusion and fission), induced mitochondrial biogenesis, preserved mitochondrial mitophagy (a mitochondrial type of autophagy that forwards the removal of injured mitochondria or mitochondria that have lost membrane potential), contributed to mitochondrial quality control (quality control refers to the combination of mitochondrial biogenesis, mitophagy and dynamics, that decisively contribute to the maintenance of appropriate cellular functioning), and protected the lung from oxidative injury induced by lipopolysaccharide (Shi et al., 2019).

#### 4.5.2 PI3K and RAS

Ang II‐induced vasoconstriction involves increases in intracellular calcium. In vascular smooth muscle cells, activation of voltage‐gated L‐type calcium channels by Ang II, which promotes calcium influx, is mediated by activation of PI3K (Quignard et al., 2001).

In an experimental model of Ang II‐induced hypertension, PI3Kgamma KO mice were protected from alterations in kidney function and renal damage, when compared with hypertensive wild‐type mice. Also, PI3Kgamma deficit abolished Ang II‐mediated renal fibroblast accretion and myofibroblast development, and inhibited collagen and extracellular matrix accumulations, as well as macrophage and T‐cell influx (An et al., 2020).

Interestingly, in a model of cerebral ischemia–reperfusion injury elicited by middle cerebral artery occlusion in the rat, AT1R blockade (losartan) annulled an elevation in inducible NOS, activated PI3K/Akt signaling thereby stimulating endothelial NOS phosphorylation and activity when compared with untreated animals subjected to cerebral ischemia/reperfusion. In addition, losartan diminished infarct sizes and enhanced neurobehavioral results in losartan‐treated versus untreated rats exposed to middle cerebral artery occlusion. This evidence underscores the benefits provided by administration of selective ARB in the setting of cerebrovascular disorders (Liu, Liu, et al., 2012).

Also, in patients with type 2 diabetic kidney disease, losartan reduced insulin resistance by attenuating oxidative stress (as assessed by determination of circulating 8‐hydroxy‐2′‐deoxyguanosine and nitrotyrosine levels) and improving insulin signaling transduction in 3T3‐L1 adipocytes through modulation of PI3K pathway, that is, by stimulating the protein expressions of phosphorylated insulin receptor, PI3K, and insulin receptor substrate 1 (Pan et al., 2015).

In cultured neural stem cells, incubation with amyloid‐β(25–35) (Aβ25‐35) oligomer inhibited cell proliferation; however, proliferation was reestablished when the cells were incubated in the presence of both Aβ25‐35 oligomer and the ARB candesartan and this effect was mediated by activation of the PI3K pathway (Choi et al., 2017).

In a model of hyperinsulinemia in the rat, administration of 15% (w/v) fructose in the drinking water during 4 weeks induced insulin resistance and reduced both AT2R neuronal function and the expression of phosphorylated Akt in dorsal root ganglia neurons. Candesartan treatment was found to ameliorate AT2R‐induced neurite outgrowth by activating the PI3K‐Akt pathway (Hashikawa‐Hobara et al., 2012).

Although high PI3K signaling is acknowledged as a characteristic cancer feature, abundant studies have revealed other unsuspected roles for the PI3K catalytic pathway in both physiological and pathological cell function (Fruman et al., 2017), which may help to reconcile the beneficial effects associated with PI3K inhibition with those related to PI3K activation described in the preceding paragraphs.

In summary, although PI3K signaling is complex and can alternatively lead to either protective or damaging actions against age‐related changes, various studies indicate that modulation of PI3K by ACEi/ARB consistently result in beneficial outcomes.

### 4.6 MAPK

In mammals, the principal components of the classical MAPK pathway include extracellular signal‐regulated kinase 1 and 2 (ERK1/2), c‐Jun N‐terminal kinase (JNK), p38, and ERK5. ERKs pathways are activated by mitotic stimuli, whereas a variety inflammatory cytokines and environmental stresses activate p38 and JNK pathways (Plotnikov et al., 2011). An atypical MAPK pathway has also been described; however, its regulation and functions are not well understood and will not be addressed here.

The MAPKs signaling pathway participates in the modulation of cell differentiation, proliferation, apoptosis, and stress response.

Whether MAPK signaling is elevated during the aging process is still a matter of debate. Nevertheless, the MAPK pathway crucially contributes to the regulation of proinflammatory cytokine synthesis, and accumulating data indicate that dysregulation of MAPK enzymes lead to the development of inflammatory disorders. In this setting, age‐related conditions—including cardiovascular disease, diabetes, neurodegenerative syndromes, and cancer—are associated with chronic moderate inflammation. Thus, it is feasible that MAPK activation is elevated during aging. This concept is supported by studies showing that in rodents several natural compounds can increase lifespan and attenuate age‐related damage accompanied with a reduction of MAPK enzyme activity, although studies in other mammalian models are lacking (Cano et al., 2019).

#### 4.6.1 MAPK and mitochondria

In human hepatoblastoma cancer cells, isoorientin—a flavonoid with anti‐inflammatory and anti‐nociceptive effects in mice—stimulates the production of ROS, which activates MAPK signaling thereby promoting mitochondria‐mediated apoptosis (Yuan et al., 2013).

Additionally, activation of murine bone marrow‐derived macrophages by treatment with LPS (lipopolysaccharide) leads to increases in the generation of mitochondrial ROS, followed by increased MAPK signaling that facilitates the macrophage response (Emre et al., 2007).

The relevance of MAPK cross talk with mitochondria, particularly in the setting of heart disease was reviewed by Javadov et al. (2014).

#### 4.6.2 MAPK and RAS

Regarding the connection between MAPKs and RAS, after binding its AT1R, Ang II strongly activates the MAPK pathway in rat neonatal heart fibroblasts (Schorb et al., 1995). Also, in vascular smooth muscle cells obtained from healthy individuals' resistance arteries, Ang II was found to activate MAPKs (p38, JNK, and ERK) by inducing the activation of NAD(P)H oxidase and the ensuing generation of ROS (Touyz et al., 2004). Furthermore, both in cultured rat vascular smooth muscle cells and in rat aorta in vivo, Ang II promoted mitochondrial ROS generation which in turn activated MAPKs, although mitochondrial ROS were not involved in Ang II‐mediated vasoconstriction (Kimura et al., 2005).

Ang II's cardiovascular, pro‐inflammatory and pro‐fibrotic actions are counterbalanced by Ang (1–7)'s anti‐inflammatory, anti‐fibrotic, and depressor effects. Changes in the relative levels of Ang II and Ang (1–7) can lead to the loss of RAS equilibrium and the consequent development of diverse conditions, such as heart failure, hypertension, atherosclerosis, myocardial infarction, among others. Interestingly, in the thoracic aortas of spontaneously hypertensive rats treated with the ARB olmesartan the ACE2 mRNA and protein contents were five times higher than in vehicle‐treated rats, whereas atenolol or hydralazine treatments had no effect, which indicates that Ang II exerts a continuous low action to reduce ACE2 (Igase et al., 2005). In this line, in cultured rat aortic vascular smooth muscle cells Ang II diminished ACE2 mRNA and activity; of note, Ang II‐induced lowering of ACE2 mRNA was prevented by a MAPK kinase inhibitor, showing that MAPK signaling plays a key role in the regulation of ACE2 in order to preserve an appropriate balance between Ang II and Ang (1–7) levels (Gallagher et al., 2008).

As a result of the above evidence showing that dysregulation of the MAPK pathway leads to inflammatory disorders and promotes mitochondria‐dependent apoptosis, and since Ang II can activate MAPK by stimulating mitochondrial ROS production and also reduce ACE2 mRNA, it can be proposed that the beneficial actions of RAS‐blockers during the aging process may be mediated by modulation of the MAPK pathway, possibly affecting mitochondria.

### 4.7 PKC

Calcium/phospholipid‐regulated protein kinases C (PKC) are a family of at least 10 serine/threonine kinases located in diverse mammalian tissues, and particularly enriched in the brain and lymphoid tissues. PKC are activated by lipid second messengers released from cell membranes after receptor‐mediated hydrolysis; thus, they convert extracellular signals into cell responses. After activation, PKC are translocated from the cytosol to other intracellular locations, a mechanism that redistributes PKC to the proximity of its substrates. PKC transduce signals that regulate the release of neurotransmitters, ion channels, receptor modulation, gene expression, synaptic plasticity, and survival (Battaini & Pascale, 2005). In consequence, normal PKC signaling is involved in the maintenance of brain health, whereas deranged PKC signaling can lead to disease. Accumulating evidence indicates that the PKC signaling pathway is altered in neuronal (Pascale et al., 2007) and nonneuronal (Corsini et al., 1999; Korzick et al., 2001) tissues of aging animals. Those age‐related changes are exemplified by the observed deficiency in PKC activation in postmortem brain samples obtained from Alzheimer's disease patients compared with age‐matched control brain samples (Wang et al., 1994). Also, the content of RACK1—a protein needed for PKC translocation—was lower in postmortem human frontal cortex samples from Alzheimer's patients as compared with age‐matched controls (Battaini et al., 1999).

The above evidence suggests that modulation of PKC signaling may turn out to be a relevant therapeutic approach to counteract different pathologies.

#### 4.7.1 PKC and mitochondria

Experimentally induced oxidative stress was found to activate PKC‐delta and to induce its translocation to the mitochondria, which was associated with the reduction of mitochondrial transmembrane potential, cytochrome c release, and apoptosis. Apoptosis was abolished when PKC activation and translocation were inhibited, indicating that the cellular apoptosis response to oxidative stress requires PKC‐delta redistribution to mitochondria (Majumder et al., 2001). Oxidant injury to renal proximal tubular cells induced the activation of PKC‐epsilon and its translocation to the mitochondria, where it reduced mitochondrial respiration, electron transport rate, and ATP production and active Na + transport (Nowak et al., 2004).

#### 4.7.2 PKC and RAS

Concerning the link between Ang II and PKC, Ang II is known to promote the proliferation of vascular smooth muscle cells partly through activation of PKC and ERK1/2 (Olson et al., 2008; Wang et al., 2008).

Heart failure, hypertension, and myocardial infarction are associated with the development of cardiac fibrosis. In this setting, activation of the RAS prompts TGF‐b1 expression that induces the transformation of fibroblasts into myofibroblasts (Berk et al., 2007). Among other remodeling factors, Ang II stimulates myofibroblast activation, which leads to increased extracellular matrix collagen production and cardiac stiffness. Significant increases in PKC‐delta and type I collagen expressions were observed in cultured rat cardiac myofibroblasts treated with Ang II or with Ang II and an AT2R blocker, when compared with cells treated with Ang II and an AT1R blocker (losartan), or Ang II and a specific PKC‐delta inhibitor (rottlerin), or Ang II and a specific siRNA for AT1R. Also, in Ang II‐infused rats, cardiac PKC‐delta and type I collagen expressions were significantly higher than in untreated controls or in rats infused with Ang II and rottlerin. These in vitro and in vivo results point to PKC‐delta as a relevant mediator in cardiac fibrosis induced by Ang II (Chintalgattu & Katwa, 2009).

Abundant studies indicate that insulin exerts relevant direct and indirect effects on the vascular endothelium through activation of the IRS/p85/PI3K/Akt signaling pathway. Those actions include arterial vasodilation mediated by activation of endothelial NOS (eNOS), as well as increases in heme oxygenase 1 (HO‐1) and vascular endothelial growth factor expression (VEGF) (Fu et al., 2021; Geraldes et al., 2008), among others. Nonetheless, activation of PKC signaling was found to inhibit the beneficial endothelial actions of insulin, thereby leading to endothelial dysfunction (Naruse et al., 2006). In this scenario, in bovine aortic endothelial cells, treatment with Ang II activated PKC signaling which inhibited insulin‐mediated eNOS activation and led to the derangement of endothelial function (Maeno et al., 2012). In Ang II‐treated cells, inhibition of insulin‐induced eNOS activation was blocked by treatment with the AT1R blocker losartan (Park et al., 2013).

In addition, in bovine adrenal glomerulosa cells Ang II increases aldosterone production through the activation of protein kinase D mediated by PKC and Src kinase family signaling (Olala et al., 2014).

In sum, altered PKC signaling is present in aging animals and can lead to disease. Also, ROS‐activated PKC‐delta reduces mitochondrial membrane potential and energy production, and Ang II induces cardiac fibrosis by activating PKC. In in vitro studies AT1R blockade prevented the damaging actions of Ang II‐induced PKC activation; therefore, it is feasible that RAS‐blockade can interfere with the aging process by counteracting the activation of PKC signaling, and potentially impacting on mitochondria.

### 4.8 Notch

The deeply pleiotropic Notch signaling pathway makes a key contribution to the communication between adjacent cells, as well as to decisions related to cell destiny. As it affects cell proliferation, differentiation, and apoptosis, it plays an important role in embryogenesis and the maintenance of adult tissue homeostasis (Kopan & Ilagan, 2009). In consequence, the dysregulation of Notch signaling participates in the pathophysiology of diverse adult diseases (Fortini, 2012).

The Notch signaling pathway is composed of four receptors (Notch 1–4), five canonical ligands, and a large group of noncanonical ligands that can activate or inhibit signaling (D'Souza et al., 2010); thus, there is an abundant number of possible receptor‐ligand combinations that points to the complexity of this signaling pathway.

Notch receptors are cell surface transmembrane proteins, containing an extra‐ and an intra‐cellular domain, and they interact with ligands (that also are cell surface transmembrane proteins) located on neighboring cells. When a ligand binds to the Notch extracellular domain in an adjacent cell, it induces Notch enzymatic cleavages and the ensuing release of Notch intracellular domain (NICD), that is translocated to the cell nucleus where it binds a transcription factor complex to finally modify target gene transcription (van Tetering & Vooijs, 2011).

In addition to the complexity of the Notch signaling pathway that derives from the large number of possible receptor–ligand combinations (as mentioned above), genetic, and genomic studies have revealed that hundreds of genes can modify Notch signals thereby increasing the intricacy of this pathway, and affecting its outcome (Louvi & Artavanis‐Tsakonas, 2012). Furthermore, depending on the cellular circumstances Notch signaling can promote contrasting responses (Balistreri et al., 2016).

Notch signaling exerts regulatory effects in the liver, skeletal muscle, brain, cardiovascular, and immune systems (Bi & Kuang, 2015); accordingly, it is not surprising that abundant evidence indicates that abnormal Notch signaling is associated with age‐related pathologies that include cardiovascular disease, neurodegeneration, Alzheimer's disease and cancer, among others (Balistreri et al., 2016).

#### 4.8.1 Notch and mitochondria

Apart from the canonical Notch functions associated with NICD's localization to the nucleus to trigger transcription, there is evidence showing that NICD can remain in the cytoplasm and interact with mitochondria. In this context, in cultured Hela, COS‐7, and HEK cell lines, expression of NICD preserved mitochondrial integrity and inhibited staurosporine‐induced apoptosis, and these effects were independent of Notch‐mediated regulation of gene transcription but relied on NICD/Akt/mitofusin (a protein involved in mitochondrial fusion) signaling (Perumalsamy et al., 2010) indicating that the signaling cascade activated by Notch regulates cell survival by interacting with mitochondrial remodeling proteins.

Additional evidence shows that Notch signaling can adjust mitochondrial metabolism in order to promote the activation of pro‐inflammatory macrophages. Macrophage activation into either pro‐inflammatory (M1‐like macrophages) or anti‐inflammatory (M2‐like macrophages) cells requires reprogramming of cell metabolism, since in M1‐like macrophages energy production relies on enhanced glucose oxidation but M2‐like macrophages are dependent on fatty acid oxidation (Lacy‐Hulbert & Moore, 2006). In vitro and in vivo studies showed that Notch 1 activation promotes macrophage transformation into the M1‐like phenotype by stimulating the expression of nuclear genes (NOS2 and pyruvate dehydrogenase phosphatase 1), as well as the expression of mitochondrial genes involved in mitochondrial oxidative phosphorylation and mitochondrial ROS production, which subsequently activate M1‐like macrophage genes. This finding is supported by observations made in a mouse model of alcoholic steatohepatitis where inhibition of Notch signaling in myeloid cells reduced the migration of circulating monocytes into the liver, the activation of M1‐like macrophages and liver inflammation (Xu et al., 2015). It should be noted that reference (Xu et al., 2015) uses the original nomenclature for M1/M2 macrophages as proposed by Mills in 2000 (reviewed in Mills (2015)). Macrophages were termed M1 and M2 to mirror the designation assigned to helper T lymphocytes (Th) into Th1 and Th2 that could be differentiated on the basis of the cytokines that they release after T lymphocyte activation. However, later research showed that in vivo an ample variety of macrophage phenotypes exists amidst M1 and M2 macrophages which revolve around the signals they were exposed to. As a result of this knew knowledge, it was recently proposed to describe macrophages as M1‐like and M2‐like instead of the original M1 and M2 terminology (Strizova et al., 2023).

Further demonstration of the link between Notch activation and mitochondrial metabolism was obtained in work with cancer cells. Thus, in triple‐negative breast cancer (TNBC), survival of cancer stem‐like cells (CSCs)—a type of cell involved in cancer development and resistance to chemotherapy—is strongly dependent on Notch signaling. In cultured TNBC cells (MDA‐MB‐231) and in a new xenograft model based on cells collected from a TNBC patient, Notch signaling was found to regulate mitochondrial metabolism and the activity of NF‐kB through a noncanonical inhibitor of nuclear factor κB kinase‐α (IKKalpha) pathway (Hossain et al., 2018).

#### 4.8.2 Notch and RAS

In HEK293 cells that express the AT1R (HEK293‐AT1 cells), treatment with Ang II was associated with increased gamma‐secretase activity; this was abolished by treatment with a gamma‐secretase inhibitor (DAPT). After binding of Notch ligands to Notch receptors located in an adjacent cell, gamma‐secretase is one of the enzymes involved in Notch receptor cleavage leading to the release of Notch intracellular domain, which thereupon is translocated to the nucleus where it modifies gene transcription. Also, DAPT treatment diminished Ang II‐mediated proliferation and migration of human aortic vascular smooth muscle cells. Furthermore, 2‐week‐Ang II infusion in wild‐type mice promoted aortic thickening of the media and perivascular fibrosis, which were attenuated by systemic administration of the gamma‐secretase inhibitor dibenzazepine. These observations indicate that Ang II ‐through its AT1R‐ activates Notch signaling consequently stimulating vascular smooth muscle cell proliferation and migration and conducting to alterations in vascular structure (Ozasa et al., 2013). The involvement of activation of Notch signaling in Ang II‐induced pulmonary artery remodeling was also observed in vessel strips obtained from rat pulmonary arteries and incubated in the presence of Ang II (Qiao et al., 2013).

Therefore, since dysregulation of Notch signaling contributes to several age‐related diseases, Ang II‐mediated AT1R activation triggers Notch signaling, and Notch was found to interact with mitochondria, it can be hypothesized that the favorable actions displayed by RAS‐blockade against aging are mediated—at least partially—by inhibition of Notch activation and the ensuing modification of gene transcription, and conceivably by acting on mitochondria.

### 4.9 Wnt

Wnt ligands—represented in humans by a 19‐member family of hydrophobic glycoproteins—are secreted into the intercellular space, and upon binding to diverse receptors located on nearby cells they activate Wnt signaling pathways leading to changes in gene transcription.

Three Wnt signaling pathways have been described: the canonical Wnt pathway (also known as Wnt/β‐catenin pathway) that is involved in the regulation of embryogenesis, cell differentiation and proliferation, tissue homeostasis, and damage repair (Clevers & Nusse, 2012); the noncanonical planar cell polarity pathway that regulates cytoskeletal architecture; and the noncanonical Wnt/calcium pathway that regulates cellular calcium levels. By acting jointly, the three pathways contribute to the regulation of cell proliferation, cell polarity, cell shape, cell survival, tissue homeostasis, and tissue design (Mikels & Nusse, 2006).

Dysregulation of Wnt signaling participates in the promotion of inflammation, fibrosis, cardiovascular disease, cancer, diabetes, neurodegeneration, all of which are associated with aging. Also, the aging process is accompanied with changes in the expression and distribution of the components of Wnt signaling in distinct tissues, leading to alterations in tissular homeostasis (Hu et al., 2020). Therefore, the Wnt signaling pathway has emerged as a relevant target to be therapeutically modulated. However, much remains to be learned about this pathway, and still unknown consequences of interfering with Wnt signaling can jeopardize the applicability of potential Wnt modulators (Maiese et al., 2008).

#### 4.9.1 Wnt and mitochondria

Interestingly, enhanced Wnt signaling was shown to firmly activate mitochondrial biogenesis and ROS production, conducting to oxidative DNA damage and expediting replicative senescence in primary mouse embryonic fibroblasts (Yoon et al., 2010). Moreover, the same authors showed that Wnt signaling induced the expression of insulin receptor substrate‐1 (IRS‐1) that straightforwardly modulated insulin signaling. This is an intriguing observation given that the insulin/IGF‐1 pathway is involved in the regulation of aging, as already mentioned in a previous paragraph.

#### 4.9.2 Wnt and RAS

Of note, in a mouse model of skeletal muscle regeneration after cryoinjury, RAS‐blockade with a subpressor dose of the ARB irbesartan enhanced muscle regeneration and function by downregulating the Wnt/β‐catenin signaling pathway (Yabumoto et al., 2015). This evidence indicates a connection between the RAS and Wnt signaling in the aging process (Kamo et al., 2016). In addition, in vitro and in vivo research showed that the Wnt/β‐catenin signaling pathway is a key regulator of the renal genes that encode RAS components (angiotensinogen, renin, ACE, and AT1R) (Zhou & Liu, 2016).

Although the number of evidences is limited, they allow to speculate that RAS‐blockade, at least partly, attenuates age‐related deterioration by reducing or suppressing Wnt signaling.

## 5 SUMMARY AND DISCUSSION

**FIGURE 1:**
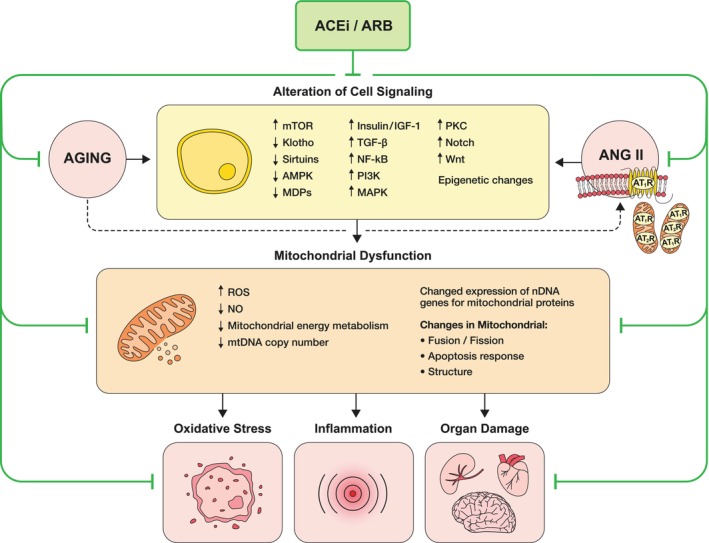
Aging is associated with changes in mTOR, AMPK, insulin/IGF‐1, TGF‐ß, NF‐kB, PI3K, MAPK, PKC, Notch, and Wnt signaling pathways. These changes contribute to mitochondrial dysfunction, that is, one of the distinctive traits of aging that finally leads to oxidative stress, inflammation, and organ damage. Ang II activates AT1 receptors thereby promoting alterations of the above mentioned age‐related signaling pathways in the same direction as the changes induced by the aging process. In addition, aging is associated with dysregulation of the renin–angiotensin system (RAS), which includes downregulation of the circulating RAS accompanied with upregulation of tissular‐RAS. Over the years, if RAS dysregulation is maintained it will generate progressive mitochondrial and organ damage, finally leading to functional alterations and aging‐associated injuries. This proposal is supported by abundant evidence showing that RAS‐blockade—with ACEi or ARB—counteracts age‐associated changes and ameliorates mitochondrial function. In addition, RAS‐blockade neutralizes the effects of RAS on mTOR, AMPK, insulin/IGF‐1, TGF‐ß, NF‐kB, PI3K, MAPK, PKC, Notch, and WNT signaling pathways. There is no direct evidence available on whether RAS‐blockers protect mitochondria by interfering with these pathways; however, as mitochondrial dysfunction strongly contributes to aging, and experimental RAS‐blockade (i) attenuates/abolishes age‐related changes and increases lifespan and (ii) protects mitochondria from the effects of aging, it can be speculated that RAS‐blockers neutralize mitochondrial dysfunction by acting on the aging regulatory pathways discussed here. ACEi, angiotensin‐converting enzyme inhibitor; AMPK, 5′ adenosine monophosphate‐activated protein kinase; Ang II, angiotensin II; ARB, angiotensin receptor blocker; IGF‐1, insulin‐like growth factor; MAPK, mitogen‐activated protein kinase; MDPs, mitochondria‐derived peptides; mtDNA, mitochondrial DNA; mTOR, mechanistic target of rapamycin; nDNA, nuclear DNA; NF‐kB, nuclear factor‐kappa B; NO, nitric oxide; PI3K, phosphoinositide 3‐kinase; PKC, calcium/phospholipid‐regulated protein kinase C; RAS, renin–angiotensin system; ROS, reactive oxygen species; TGF‐b, transforming growth factor beta; Wnt, Wingless and int‐1.

The evidence summarized here (Figure 1) shows that the aging regulatory pathways mTOR, AMPK, insulin/IGF‐1, TGF‐b, NF‐kB, PI3K, MAPK, PKC, Notch, and Wnt affect mitochondrial function. In addition, by acting on its various receptors, Ang II—RAS's main effector—stimulates/inhibits the above mentioned pathways and impact on mitochondria. Aging is associated with alterations in these signaling pathways, thus contributing to mitochondrial dysfunction, that is, one of the distinctive traits of aging. The aging process is also associated with dysregulation of the RAS, which includes downregulation of the circulating RAS accompanied with upregulation of tissular RAS. Therefore, it is feasible that age‐related RAS derangements contribute to aging by activating these signaling pathways, thereby negatively impacting on mitochondria, conducting to mitochondrial/cellular oxidative stress, and activating inflammatory processes. If the last mentioned state is maintained over the years it will conduct to progressive organ changes, finally leading to functional alterations and aging‐associated injuries. RAS‐blockade has been variously reported to counteract the latter effects, although there is no direct evidence available on [RAS/RAS‐blockade‐aging regulatory pathway] and mitochondria interactions. However, as mitochondrial dysfunction strongly contributes to aging, and experimental RAS‐blockade (i) attenuates/abolishes age‐related changes and increases lifespan and (ii) protects mitochondria from the effects of aging, it can be speculated that RAS‐blockers neutralize mitochondrial dysfunction by acting on the aging regulatory pathways discussed here.

We acknowledge that the above signaling pathways are regulated in a complex fashion, that in many cases the outcome of their activation is dependent on cellular context, that different pathways engage in cross talk, and that a great deal still needs to be learned in order to obtain a clear picture. In consequence, although the available evidence allows to propose that the favorable effects of RAS‐blockade against aging might be related to the amelioration/normalization of age‐related signaling pathways, we are still far from being able to describe with certitude those connections. However, these shortcomings do not tarnish the astounding number of results that point to a beneficial role of RAS‐blockade in aging and age‐related disease.

The amount of information available prevented us from citing all articles; therefore, we commented only on the number of examples that we considered that could clarify the point.

Although several behavioral interventions—such as calorie restriction (CR), intermittent fasting, moderate exercise, or plant‐based diets—were shown to modulate the rate of aging, adherence to these lifestyles is hard to attain in humans. As CR is the most robust intervention to extend health‐ and lifespan, abundant efforts were dedicated to discover compounds that induce CR‐similar actions. Among these CR‐mimetics, rapamycin and metformin are the most studied agents, which are FDA‐approved for other indications. However, both compounds are associated with adverse effects that may restrict their use as antiaging interventions in healthy subjects (Martel et al., 2021).

In recent years an increasing body of evidence has appeared regarding the renal and cardiovascular protection that the sodium‐glucose cotransporter‐2 inhibitors (SGLT‐2i or gliflozins) provide, along with an impressive reduction in mortality in patients with cardiovascular and renal disease with or without type 2 diabetes. These drugs have a remarkable effect at the mitochondrial level, in many ways similar to that of CR and complementary to RAS‐blockade. As a result, their potential favorable effect on aging is highly probable (Hoong & Chua, 2021; Sanz et al., 2023; Scisciola et al., 2023). In any case, in order to qualify as candidates for use against human aging, this family of drugs still needs both proof of safety after prolonged use, and cost reduction.

ACEi and ARB are widely prescribed for the treatment of hypertension (NIfHaCENHiadam, 2011), diabetic microalbuminuria, or proteinuric renal disease (Disease NIfHaCENMoCK, 2014), heart failure (Management NIfHaCENChfia, 2010), and after myocardial infarction (Prevention NIfHaCENMis, 2013). Discontinuation of ACEi/ARB treatment can occur due to adverse effects that include either elevation of serum creatinine of 30% or more (observed in 1.7% or 1.2% of patients), or serum potassium >6 mmol/L (reported in 0.4% of patients) (Schmidt et al., 2017a, 2017b) hypotension, dry cough [observed in 5%–35% of patients receiving ACEi (Bezalel et al., 2015)] or angioedema (up to 0.7% with ACEi, and much less frequently with ARB (Bezalel et al., 2015; Vleeming et al., 1998)).

However, it should be taken into account that (a) to potentially slow down the rate of aging in humans ACEi or ARB would be administered to healthy patients, and (b) it is feasible that ACEi or ARB doses lower that those used therapeutically would be needed, since—as reported above—treatment of spontaneously hypertensive rats (Ferder et al., 1998) with a non‐antihypertensive dose of the ACEi enalapril was associated with protective tissue and mitochondria actions in the heart.

## 6 CONCLUSION

In view of the above evidence as a whole, it seems reasonable to propose that the foundation is laid for conducting clinical trials aimed at testing whether ACEi and ARB treatments offer the possibility to live longer and in better health. It is interesting to note that, since ACEis and ARBs are low cost and well‐tolerated therapies already in use for more than 35 years, redirecting their administration to attenuate/prevent the effects of aging seems to be devoid of major difficulties.

### 6.1 Perspectives and significance

Humankind is experiencing a rise in longevity (D'Souza et al., 2010), which is widely considered to be a medical advance as long as it is accompanied with the improvement of healthspan. Here we have reviewed RAS inhibitor's positive impact on multiple aging regulatory pathways, including their relation with mitochondrial protection.

In addition, solid evidence indicates that RAS‐blockade—even at subclinical levels—attenuates age‐related decline and increases rodent lifespan. In humans, RAS‐blockers were shown to exert reno‐ and cardioprotective actions outside the limits of blood pressure control (Louvi & Artavanis‐Tsakonas, 2012; van Tetering & Vooijs, 2011), and they are associated with good tolerability and safety (Balistreri et al., 2016). As a result of the above substantial knowledge, a logical next step would be to undertake well‐designed clinical trials to investigate whether long‐term low‐level RAS‐blockade is able to prolong human life while reducing age‐associated disease.

## AUTHOR CONTRIBUTIONS

**Elena M. V. de Cavanagh**: Conceptualization, writing—original draft, and writing—review and editing; **Felipe Inserra**: Conceptualization and writing—review and editing; **León Ferder**: conceptualization and writing—review and editing.

## FUNDING INFORMATION

This article did not receive any specific grant from funding agencies in the public, commercial, or not‐for‐profit sectors.

## CONFLICT OF INTEREST STATEMENT

The authors have no conflict of interest to declare.

## ETHICS STATEMENT

None.
